# Real-time three-dimensional transesophageal echocardiography to predict artificial chordae length for mitral valve repair

**DOI:** 10.1186/1749-8090-8-137

**Published:** 2013-05-30

**Authors:** Huan-lei Huang, Xu-jing Xie, Hong-wen Fei, Xue-jun Xiao, Jing Liu, Jian Zhuang, Cong Lu

**Affiliations:** 1 The Department of Cardiovascular Surgery, Guangdong Provincial Cardiovascular Institute, Guangdong General Hospital, Dongchuan Road, Guangzhou, 510100, China; 2 The Department of Cardiology, The Third Affiliated Hospital of Sun Yat-sen Universty, Tianhe Road, Guangzhou, 510630, China; 3 The Department of Cardiology, Guangdong Provincial Cardiovascular Institute, Guangdong General Hospital, Dongchuan Road, Guangzhou, 510100, China

**Keywords:** Artificial chordae replacement, Mitral valve repair, Real-time three-dimensional transesophageal echocardiography

## Abstract

**Background:**

Artificial chordae replacement is an effective technique for mitral valve repair, however, it is difficult to accurately determine the length of artificial chordae. This study aimed to assess the reliability and accuracy of real-time three-dimensional transesophageal echocardiography (TEE) to predict the length of artificial chordae preoperatively.

**Methods:**

From December 2008 to December 2010, 48 patients with severe mitral regurgitation successfully underwent mitral valve repair using artificial chordae replacement. The patients were divided into a TEE pre-measurement group (n = 26) and a direct measurement group (n = 22), according to the method used to determine the length of artificial chordae. Cardiopulmonary bypass time, aortic cross-clamp time, and the recurrence rate of moderate or severe mitral regurgitation were compared between the two groups.

**Results:**

There were no operative deaths in either group. The mean cardiopulmonary bypass time was 113.0 ± 18.7 min and 127.0 ± 28.9 min (p < 0.05), and the aortic cross-clamp time was 70.0 ± 16.6 min and 86.0 ± 20.7 min (p < 0.05) in the TEE pre-measurement group and direct measurement group, respectively. The difference between the pre-measured artificial chordal length and actual constructed artificial chordal length was not significant in the TEE pre-measurement group (p > 0.05). Although the difference in the incidence of moderate or severe mitral regurgitation between the two groups was not significant (p > 0.05), the incidence in the TEE pre-measurement group (3.8%) was lower than that in the direct measurement group (18.2%).

**Conclusions:**

Real-time three-dimensional transesophageal echocardiography can accurately predict the length of artificial chordae required for mitral valve repair, and shortens cardiopulmonary bypass time and aortic cross-clamp time while improving the results of mitral valve repair.

## Background

The combination of ring annuloplasty with leaflet quadrangular or triangular resection and reconstruction has become the standard technique for posterior mitral leaflet prolapse [1]. However, problems remain in the ability to repair anterior leaflet prolapse and more complex pathologies, such as Barlow’s Disease. Patients with anterior leaflet prolapse and complex mitral lesions often undergo valve replacement in less-experienced centers [2]. Artificial chordae replacement is an effective technique for treating mitral regurgitation (MR) resulting from anterior and/or posterior leaflet prolapse without the need for quadrangular or triangular resection, which reduces the effective area of coaptation. The good long-term outcomes of artificial chordae replacement are also attributed to the fact that it achieves the largest possible mitral orifice area and area of leaflet coaptation [3,4]. However, artificial chordae replacement is technically more demanding. The most intriguing challenge in artificial chordae replacement is how to determine the appropriate length of the artificial chordae and maintain the length unchanged when the polytetrafluoroethylene (PTFE) (Gore-Tex) sutures are tied [5]. Various techniques have been used to determine the length of the artificial chordae, including: the hydrostatic test [6], direct measurement of normal chordae length [7], and using the distance from the papillary muscle (PM) tip to the corresponding annulus as the artificial chordae length [8]. Since these methods are performed in the arrested heart, it is difficult to determine the precise length of artificial chordae, because the geometry of the left ventricle is different in the arrested heart compared with that in the beating heart [9]. Another potential disadvantage of direct measurement is that it is time consuming. Therefore, none of these techniques is entirely satisfactory [10,11]. Mandegar and colleagues [12] preoperatively determined the artificial chordae length using two-dimensional transesophageal echocardiography; the measurement of the distance between the head of the posterior PM and the mitral annulus plane at coaptation of the leaflets was regarded as the artificial chordae length. However, the coaptation of the mitral leaflets generally occurs below the annular plane, and it was argued that their method may produce variable results [13]. In this study, the distance from the PM tip to the normal closing point of corresponding leaflet segment was measured preoperatively using real-time three-dimensional transesophageal echocardiography (RT3D-TEE), and this method was used to determine the length of the neo artificial chordae to be implanted. We believe that this technique is more reliable, accurate and saves time compared with other techniques.

## Methods

### Patients

The informed consent obtained from all patients. The study was approved by the ethics committee of Guangdong General Hospital. Between December 2008 and December 2010, 48 patients were enrolled in this retrospective study. The selection criteria included successfully undergoing isolated mitral valve repair for mitral valve prolapse and with artificial chordae replacement. All the patients had concomitant implantation of an annuloplasty ring (C-E physio, Edwards Lifesciences LLC, USA), and the mitral valve repair procedures were performed by the same surgeon at Guangdong Provincial Cardiovascular Institute. Patients who underwent concomitant procedures were excluded. Patients’ medical records were reviewed after discharge to collect preoperative, intraoperative and postoperative data.

**Figure 1:**
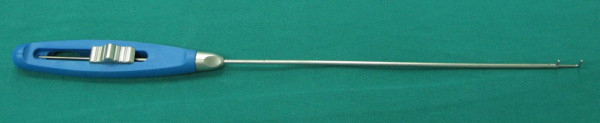
Custom made caliper (Landanger Inc., France) that was used to measure the length of the normal native chordae.

The patients were divided into two groups according to their willingness to undergo real-time three-dimensional transesophageal echocardiography (RT3D-TEE) before surgery. There were 26 patients who consented to RT3D-TEE in the ultrasound room preoperatively, mitral valve quantification had been performed using Q-LAB 6.0 software (Philips Company, USA) to pre-measure the length of artificial chordae before patients underwent surgery, and these patients were included in the TEE pre-measurement group. Twenty-two patients who refused preoperative RT3D-TEE were included in the direct measurement group. In these patients, the normal chordae length of the near leaflet segment was directly measured in the arrested heart during surgery using a custom made caliper (Landanger Inc., France) (Figure 1), and this value was used as the target length of the neochordae to be implanted.

The indication for surgery was symptomatic severe mitral insufficiency. The severity of MR was assessed quantitatively according to the area of the MR jet and area of left atrium in the four-chamber view. The following grading of MR was used [14]: grade 0, no MR; grade 1, mild (MR jet area/left atrium area < 20%); grade 2, moderate (MR jet area/left atrium area 20-40%); grade 3, severe (MR jet area/left atrium area 41-60%); and grade 4, MR jet area/left atrium area >60%.

### Echocardiography scan

**Figure 2:**
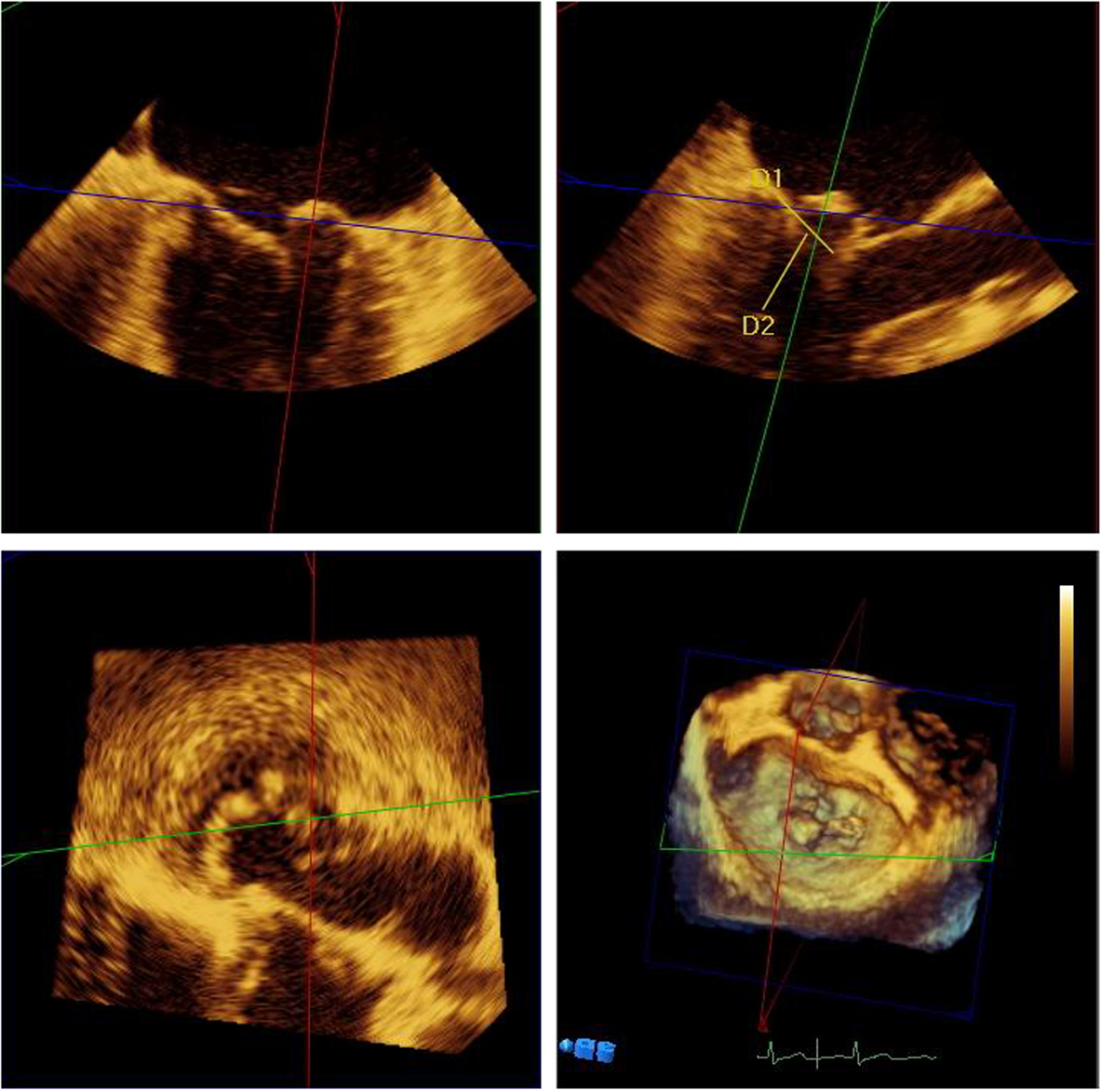
**D2 is the length of artificial chordae to be implanted.** In the 3D zoom view that includes the mitral valve leaflet,chordae tendineae and papillary muscle at the systolic phase. The X-plane and Y-plane were changed to show the relationship between the prolapsed segment and papillary muscle in the Z-plane using mitral valve quantification software. The distance (D2) from the tip of the papillary muscle to the normal closing point of the leaflet segment was regarded as the reference length of artificial chordae.

**Figure 3:**
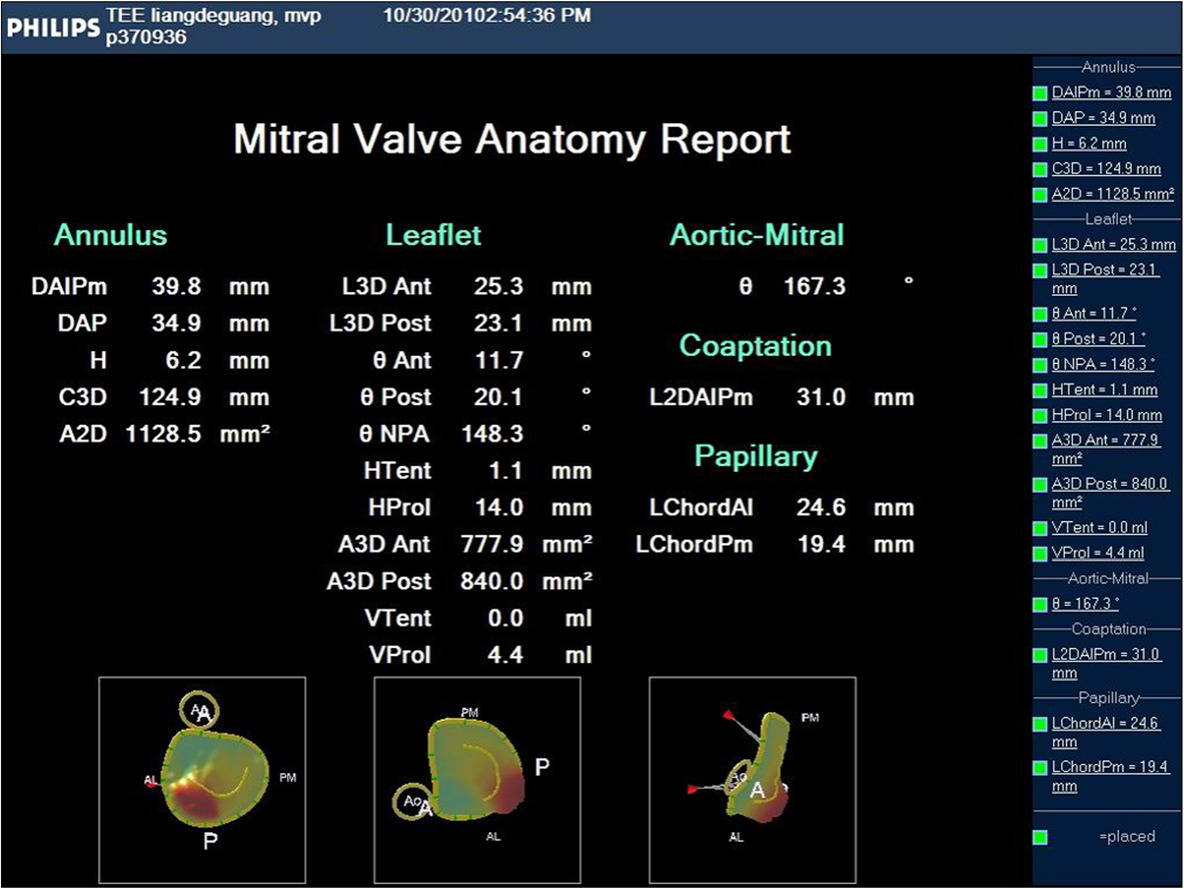
Results of the mitral valve analysis provided the length of the artificial chordae tendineae to be planted.

RT3D-TEE (Philips IE33, Philips Company, USA) was performed with a TEE transducer (X7-2t, Philips Company, USA). After obtaining informed consent, patients in the TEE pre-measurement group underwent preoperative and intraoperative RT3D-TEE. Data on the surface area and altitude of the prolapsed segment acquired by RT3D-TEE was used to help plan the surgical intervention. The length of artificial chordae was pre-measured using mitral valve quantification software (Q LAB 6.0, Philips Company, USA). The method of pre-measuring the length of artificial chordae with RT3D-TEE was as follows: after obtaining a 3D zoom view of the mitral valve including the valve leaflet, chordae tendineae and PM at the systolic phase, the X-plane and Y-plane were changed to show the relationship between the prolapsed segment and PM in Z-plane using the mitral valve quantification software, the distance (D2) from the PM tip to the expected coaptation surface of corresponding leaflet segment was considered the reference length of artificial chordae (Figure 2). Figure 3 shows the anatomy of the mitral valve; the analytic results indicated the length of the artificial chordae. In the TEE pre-measurement group,the involved prolapse segment was detected first. If A1, P1 segment prolapsed, the distance from the expected coaptation surface to the anterior PM was measured as the reference length of the neochordae; if A3, P3 segment prolapsed,the distance from the expected coaptation surface to the posterior PM was measured; and if A2, P2 segment prolapsed, the distance from the expected coaptation surface to the nearest PM was measured; So, the neochordae of different segments had specific pre-measured length. The pre and intra-operative echocardiographic exam was taken by the same echocardiographic operator. It took about 10 min to acquire 3D-TEE images per patient, and 10 ~ 15 min to estimate the length of artificial chordae using mitral valve quantification software.

Patients in the TEE pre-measurement group were examined a second time using RT3D-TEE before they were weaned from cardiopulmonary bypass to identify the competency of the mitral valve and measure the length of constructed artificial chordae.

### Operative techniques

**Figure 4:**
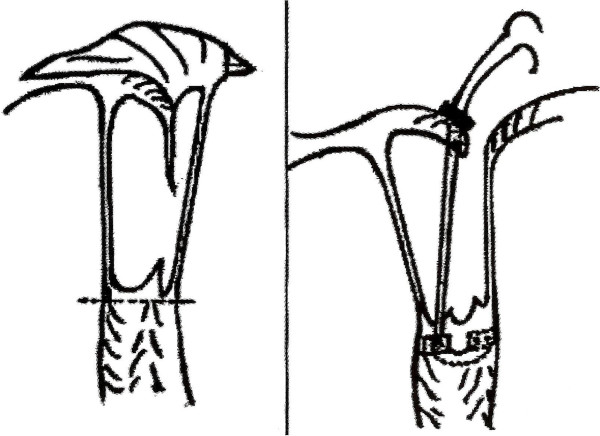
**A titanium clip was placed at the marked point of the PTFE suture at the atrial side of the leaflet.** The distance from the point of the papillary muscle tip to the marked point equals the pre-measured length of the neochordae.

All the mitral valve repair procedures were performed by the same consultant surgeon and his team. Before implantation of artificial chordae and a full annuloplasty ring, 2–0 dacron sutures were placed around the mitral valve annulus. Saline was injected into the left ventricle to evaluate the severity of regurgitation of the prolapsed segment. A double-armed 4–0 polytetrafluoroethylene (PTFE; Gore-Tex, W.L. Gore & Associates, Inc, USA) pledgetted suture was passed through the fibrous tip of the PM, to which the prolapsed leaflet segment was attached and tied with a second pledget on the other side of the PM. The two arms of the stitch were then passed through the free edge of the prolapsed segment of the mitral valve from the ventricular side to the atrial side. The length of suture was marked according to the value measured preoperatively by RT3D-TEE in TEE pre-measurement group. Then, a titanium clip was placed at the marked point of the suture on the atrial side of the leaflet (Figure 4). The left ventricle was again inflated with saline to re-evaluate residual regurgitation if any, and the two arms of the suture were tied. More PTFE chordae were implanted using the same method if needed. The number of artificial chordae depended on area of the prolapsed leaflet segment and the number of ruptured chordae tendineae. After artificial chordae implantation, an appropriate size annuloplasty ring was implanted and fixed with the previously-placed sutures. The size of the annuloplasty ring was determined according to the intraoperative measurement of patient’s anterior leaflet surface with sizers. In patients in the direct measurement group the chordae length was measured in the arrested heart using a custom made caliper (Landanger Inc., France). The method of implantation of neochordae in the direct measurement group was the same as that in the TEE pre-measurement group. Cardiopulmonary bypass time, aortic cross-clamp time and the recurrence rate of moderate or severe MR were compared between the two groups. In patients in the TEE pre-measurement group, the pre-measured length and the actual length of artificial chordae was compared (p > 0.05).

### Follow- up evaluation

Patients were followed-up by Guangdong Provincial Cardiovascular Institute Valve Clinic. Regular outpatient visits included clinical assessment and echocardiographic evaluation. The follow up rate was 100%; the mean follow-up interval was 12.6 ± 8.4 months (range, 2–36 months). Echocardiography was performed by two specified cardiologists at 3-, 6- and 12-month intervals.

### Data analysis

Continuous data are presented as the mean ± SD, whereas categorical data are presented as frequency and percentages. Data analysis was carried out in SPSS 13.0 (SPSS Inc, Chicago, Ill) software. Numeration data was compared between groups using Pearson’s χ^2^ test or Fisher’s exact test. An independent-samples t-test was used to compare continuous variables, such as age, area of the MR jet, number of artificial chordae, time of cardiopulmonary bypass, time of aortic cross-clamp and the area of the residual MR jet. Significant statistical differences were considered to exist if p < 0.05.

## Results

**Figure 5:**
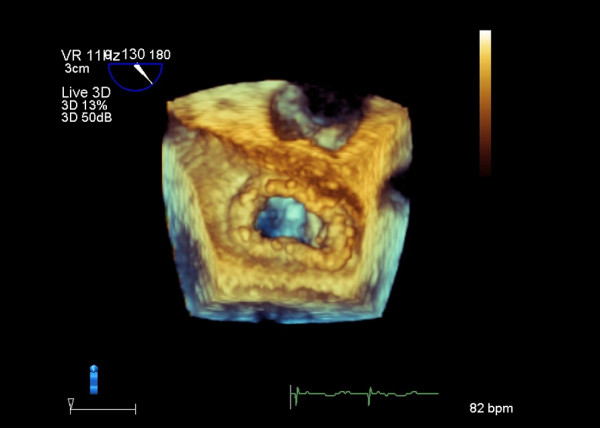
**Mitral valve repair was performed with artificial chordae and annuloplasty ring implantation.** RT3D-TEE image shows the mitral valve closure in well condition.

There were 35 males and 13 females in this study. The median age at operation was 47 years (12–68 years). Anterior leaflet lesions were present in 14 patients, posterior leaflet lesions in 24 patients, bileaflet lesions in 9 patients and a commissural lesion in 1 patient. Causes of MR included mucoid degeneration in 36 patients with elongated and/or ruptured chordae, Barlow’s disease in 5 patients with elongated chordae and enlarged mitral annulus, congenital abnormality in 4 patients with elongated chordae and leaflet cleft, rheumatic heart disease in 2 patients with elongated and thickened chordae, and infective endocarditis in 1 patient with ruptured chordae and leaflet perforation. There was no significant difference in age, the leaflet involved, the etiology of MR or severity of MR between the TEE pre-measurement group and the direct measurement group, and the complex etiologies of MR (e.g. Barlow’s disease, endocarditis, and rheumatic disease) were comparable between the two groups. The mean grade of MR was 3.2 ± 0.5 in the TEE pre-measurement group and 3.3 ± 0.6 in the direct measurement group (p > 0.05). There were no operative deaths in either group. The time of cardiopulmonary bypass (CPB) was 113.0 ± 18.7 min and 127.0 ± 28.9 min (p < 0.05), and the time of aortic cross-clamp (ACC) was 70.0 ± 16.6 min and 86.0 ± 20.7 min (p < 0.01) in TEE pre-measurement group and the direct measurement group, respectively. The number of artificial chordae per patient was 2.0 ± 0.8 (range, 1–4) in TEE pre-measurement group, and 1.7 ± 0.6 (range, 1–3) in the direct measurement group. The comparison of patients’ characteristics between the TEE pre-measurement group and the direct measurement group was shown in Table 1. In the TEE pre-measurement group, the pre-measured artificial chordal length of the anterior leaflet was 21.3 ± 2.8 mm, and the actual constructed artificial chordal length was 20.8 ± 2.3 mm. The pre-measured artificial length of the posterior leaflet was 20.0 ± 2.1 mm, and the actual constructed artificial chordal length was 19.5 ± 2.0 mm. The difference between the pre-measured length and actual length of the two leaflets was not significant (p > 0.05). Figure 5 was a RT3D-TEE image of post-operative mitral valve reconstruction with the new chordae, which showed the mitral valve closure in well condition. During the follow-up period, there were no late deaths in either group. In the TEE pre-measurement group, trace MR was detected in 7 patients, mild MR in 4 patients, moderate MR in 1 (3.8%) patient and severe MR in no patients. In the direct measurement group, trace MR was detected in 7 patients, mild MR in 2 patients, moderate MR in 3 (13.6%) patients and severe MR in 1 (4.5%) patient, who refused reoperation. Although the difference in the incidence of moderate or severe MR between the two groups was not significant (p > 0.05), the incidence in the TEE pre-measurement group (3.8%) was lower than that in the direct measurement group (18.2%). There were no documented artificial chordae ruptures in either group. Table 2 demonstrated the results of surgery in two groups and the comparison between pre-measured length and actual constructed artificial chordal length in TEE pre-measurement group.

**Table 1:** Characteristics of patients in TEE pre-measurement group and direct measurement group

	**TEE pre-measurement group**	**Direct measurement group**	***P-*value**
Number of patients	26	22	
Male: female ratio	18:8 (69%:31%)	17:5 (77%:23%)	0.532
Age (years)	45 ± 16	40 ± 18	0.236
Leaflet involved			0.627
Anterior leaflet	8 (31%)	6 (27%)	
Posterior leaflet	14 (54%)	10 (45%)	
Bi-leaflet	4 (15%)	5 (23%)	
Commissure	0	1 (5%)	
Etiology of MR			0.858
Mucoid degeneration	20 (77%)	16 (73%)	
Barlow's disease	3 (11.5%)	2 (9%)	
CHD	2 (7.7%)	2 (9%)	
RHD	1 (3.8%)	1(4.5%)	
SBE	0	1(4.5%)	
Preoperative AMJR (cm^2^)	14.8 ± 3.9	15.0 ± 5.0	0.729
Preoperative MR grade	3.2 ± 0.5	3.3 ± 0.6	0.844

*TEE* transesophageal echocardiography, *MR* mitral regurgitation, *CHD* congenital heart disease, *RHD* rheumatic heart disease, *SBE* subacute bacterial endocarditis, *AMRJ* area of mitral regurgitation jet.

**Table 2:** The surgical results and the comparison between pre-measured length and actual length of neochordae

	**TEE pre-measurement group**	**Direct measurement group**	***P-*value**
Time of CPB (min)	113.0 ± 18.7	127.0 ± 28.9	0.045
Time of ACC (min)	70.0 ± 16.6	86.0 ± 20.7	0.000
Number of artificial chordae	2.0 ± 0.8	1.7 ± 0.6	0.131
Pre-measured neochordae length of anterior leaflet(mm)	21.3 ± 2.8 mm		0.608
Actual neochordae length of anterior leaflet(mm)	20.8 ± 2.3 mm		
Pre-measured neochordae length of posterior leaflet(mm)	20.0 ± 2.1 mm		0.385
Actual neochordae length of posterior leaflet(mm)	19.5 ± 2.0 mm		
Moderate or severe MR during follow-up	1 (3.8%)	4 (18.2%)	0.252

*TEE* transesophageal echocardiography, *CPB* cardiopulmonary bypass, *ACC* aortic cross clamp, *MR* mitral regurgitation.

## Discussion

Mitral valve leaflet rectangular resection and reconstruction plus annuloplasty ring implantation usually has excellent results for patients with isolated posterior mitral leaflet prolapse, but is not as satisfactory for complex lesions, such as Barlow’s disease and anterior leaflet prolapsed. In these latter two conditions, re-operation rate varies from 5% to 20% [15,16]. Mitral valve repair by replacement of chordae tendineae with PTFE sutures expands the indication and improves the long-term outcomes of mitral valve repair [5,17]. In this study, 47.9% of the patients had anterior leaflet prolapse and some of them would have undergone mitral valve replacement if the artificial chordae replacement technique was not applied.

We used RT3D-TEE to pre-measure the length of artificial chordae preoperatively with the heart beating rather than arrested. The length of artificial chordae measured by preoperative RT3D-TEE was almost identical to the length of the implanted artificial chordae, because the preoperative length was measured under physiological loading conditions. This more accurate assessment of the artificial chordae length should result in complete coaptation of the leaflets and improved early and mid-term results. Weber and colleagues [18] reported that the length of virtual neochordae measured in systole was different from that measured in diastole. The most common technique is the direct measurement of the distance from the PM tip to the corresponding annulus in arrested heart, the geometry of the left ventricle in the arrested heart is similar to that in diastole. However, the distance between the tip of the PM and the level of the annulus is variable in diastole and systole. The length pre-measured in systole with echocardiography will be more accurate than that directly measured in arrested heart for constructing artificial chordae in mitral valve repair. On the other hand, in the direct measurement group, the cardiac surgeon had to spend additional time to directly measure the length of normal chordae tendineae and repeatedly adjust the length of the neochordae. This significantly prolonged the time of cardiopulmonary bypass and aortic cross-clamp. Our findings suggested the RT3D-TEE was a simple and reliable method for predicting the appropriate length of artificial chordae, and this method was helpful to reduce the cardiopulmonary bypass time and aortic cross-clamp time, and improve the outcomes of mitral valve repair with artificial chordae implantation. Canna et al. [19] reported that RT3D-TEE provided more accurate mapping of mitral valve prolapse than two-dimensional imaging, since it provided anatomical details of the mitral valve. Pathologic processes (e.g. chordal rupture) could be quickly identified and precisely localized with the aid of RT3D-TEE [20,21]. The tip of the PM, elongated or ruptured chordae and the corresponding leaflet segments could be correctly located using RT3D-TEE. Complete information on the leaflet lesion was provided to the surgeon before the heart was opened.

Maintaining the length of the artificial chordae constant during tying the two arms of PTFE suture is another key to surgical success. Because PTFE sutures are soft and slippery, the knot slides easily. We coped with this problem by using a titanium clip to fix the marked point on the suture (Figure 4), as has been used by other investigators. It was also reported that the problem could be overcome with the ‘adjustable’ artificial chordal replacement technique [5].

Compared with two-dimensional echocardiography, RT3D-TEE not only improves the morphologic evaluation of the mitral valve [22], but also allows quantitative evaluation of the mitral annulus, prolapsing segment and length of chordae. 3D echocardiography can easily locate the heads of both PMs, and the 3D measurement can be useful at the time of mitral valve repair when additional subvalvular techniques are used [23,24]. Our results suggested that RT3D-TEE was accurate for predicting the appropriate length of artificial chordate, which should allow the surgeon to determine the length of artificial chordae to be implanted before the patient enters the operating room. This should result in greater operator confidence to adequately perform mitral valve repair with artificial chordae replacement. Three-dimensional echocardiography provides an anatomic view of the mitral valve that is similar to that seen by the surgeon after atriotomy [25,26]. It has become the most promising technique for planning, surgical optimization and postoperative surveillance of mitral valve repair [27]. Furthermore, RT3D-TEE is also useful for percutaneous mitral valve repair using the edge-to-edge technique [28].

## Conclusions

In conclusion, artificial chordal replacement was found to be an effective method for the repair of both a prolapsing anterior leaflet and/or posterior leaflet and provided satisfactory early and mid-term results in this study. RT3D-TEE can supply dynamic three-dimensional images of the mitral valve, and can accurately quantitate the mitral valve lesion and exactly predict the length of the artificial chordae preoperatively. The pre-measurement of the length of the artificial chordae using RT3D-TEE is a reliable, accurate and time-saving method for use in mitral valve repair.

## Abbreviations

MR: Mitral regurgitation; PTFE: Polytetrafluoroethylene; PM: Papillary muscle; RT3D-TEE: Real-time three-dimensional transesophageal echocardiography; CPB: Cardiopulmonary bypass; ACC: Aortic cross-clamp.

## Competing interests

The authors declare that they have no competing interests*.*

## Authors’ contributions

LC and HHL participated in the mitral valve repair. HHL and XXJ drafted the manuscript and performed the statistical analysis. FHW carried out the examination of echocardiography. XXJ and ZJ participated in the design of the study. LC and LJ conceived of the study, and participated in its design and coordination and helped to draft the manuscript. All authors read and approved the final manuscript.
